# Exploring the potential of endophyte-plant interactions for improving crop sustainable yields in a changing climate

**DOI:** 10.3389/fpls.2024.1349401

**Published:** 2024-03-20

**Authors:** Lorenzo Sena, Erica Mica, Giampiero Valè, Patrizia Vaccino, Nicola Pecchioni

**Affiliations:** ^1^ Dipartimento di Scienze della Vita, Sede Agraria, UNIMORE - Università di Modena e Reggio Emilia, Reggio Emilia, Italy; ^2^ Centro di Ricerca Cerealicoltura e Colture Industriali, CREA – Consiglio per la Ricerca in Agricoltura e l’Analisi dell’Economia Agraria, Vercelli, Italy; ^3^ Dipartimento per lo Sviluppo Sostenibile e la Transizione Ecologica, UPO – Università del Piemonte Orientale, Complesso San Giuseppe, Vercelli, Italy; ^4^ Centro di Ricerca Cerealicoltura e Colture Industriali, CREA – Consiglio per la Ricerca in Agricoltura e l’Analisi dell’Economia Agraria, Foggia, Italy

**Keywords:** endophytes, climate change, stress tolerance, omic techniques, arbuscular mycorrhizal fungi, microbiota

## Abstract

Climate change poses a major threat to global food security, significantly reducing crop yields as cause of abiotic stresses, and for boosting the spread of new and old pathogens and pests. Sustainable crop management as a route to mitigation poses the challenge of recruiting an array of solutions and tools for the new aims. Among these, the deployment of positive interactions between the micro-biotic components of agroecosystems and plants can play a highly significant role, as part of the agro-ecological revolution. Endophytic microorganisms have emerged as a promising solution to tackle this challenge. Among these, Arbuscular Mycorrhizal Fungi (AMF) and endophytic bacteria and fungi have demonstrated their potential to alleviate abiotic stresses such as drought and heat stress, as well as the impacts of biotic stresses. They can enhance crop yields in a sustainable way also by other mechanisms, such as improving the nutrient uptake, or by direct effects on plant physiology. In this review we summarize and update on the main types of endophytes, we highlight several studies that demonstrate their efficacy in improving sustainable yields and explore possible avenues for implementing crop-microbiota interactions. The mechanisms underlying these interactions are highly complex and require a comprehensive understanding. For this reason, omic technologies such as genomics, transcriptomics, proteomics, and metabolomics have been employed to unravel, by a higher level of information, the complex network of interactions between plants and microorganisms. Therefore, we also discuss the various omic approaches and techniques that have been used so far to study plant-endophyte interactions.

## 1 Introduction

The main objective of this review is to to provide a summarized update of the existing knowledge about the interactions between plants and endophytic microorganisms, focusing on their potential for improving crop production and resilience. With this aim, we reviewed their potential role for facing key challenges, such as biotic and abiotic stresses, in the context of climate change. Among the plant-microorganisms interactions, we have restricted our analysis to the endophytic ones, and among these, to the ones between endophytes and agricultural plants.

Before analysing the impact of the endophytic interactions on the stress resiliences, enclosing in a wider view the plant mineral nutrition and its relationships with quality, we have first defined the scenario of climate change in which the microbiota can have a renewed role and importance. Then, we identified the boundaries and characteristics of the endophytes within the microbiota, the general mechanisms and ontogenesis of their interactions with agricultural plants, as well as their classification in groups significant for the agricultural systems. We also explored the relevant ‘omic’ techniques as essential tools for analysing complex microbial communities and their interactions with plants. Omics are a natural choice for studying the complex symbiosis between plants and microorganisms in view of their exploitation (Plett and Martin, 2018; Sharma et al., 2020; Sandrini et al., 2022). Most papers analysed in this review are recent reviews and original papers published in the last decade, in particular the original research papers. Databases used during the research process included PubMed (https://pubmed.ncbi.nlm.nih.gov/), Litmaps (https://www.litmaps.com/), Open Knowledge Maps (https://openknowledgemaps.org/). Keywords used during the first phase of information collection comprehended: endophytes, abiotic stress, biotic stress, omics, PGPM. Several reviews have examined the interaction between plants and Plant Growth Promoting Microorganisms (PGPMs) (Vandenkoornhuyse et al., 2015; Santoyo et al., 2016; Khatoon et al., 2020), including potential benefits in biotic and abiotic stress scenarios (Miliute et al., 2015; Yan et al., 2019; Kamran et al., 2022), and have been here considered and updated. Also, the impact of climate change has been thoroughly studied and reviewed (Driga and Drigas, 2019; Lynch et al., 2021), as it has specific aspects for plant and microorganism interactions (Compant et al., 2010; Trenberth et al., 2014; Classen et al., 2015; Shahzad et al., 2021), and both these fields have been here contextualized with respect to topics treated in this review. Abiotic stressors such as drought, heat, and salinity have been included since of highly significant concern (Lipiec et al., 2013; Evelin et al., 2019; Ma et al., 2020; Angon et al., 2022). The role of microorganisms in protecting from biotic stresses (Pandey et al., 2019; Chaudhary et al., 2022), including viruses (Bao and Roossinck, 2013), nematodes (Bernard et al., 2017; Gamalero and Glick, 2020; Pulavarty et al., 2021), insects (Bradshaw et al., 2016), fungi, and bacteria (Muthu Narayanan et al., 2022) have been previously discussed and are here gathered and updated.

In this review, we combined various aspects, usually considered individually, that characterize the symbiosis between endophytes and plants: mechanisms of selection and interaction, effects on biotic and abiotic stress factors, omic techniques for the study of such complex symbioses, consequences from the nutritional point of view of the interaction between endophytes and agricultural crops. For each type of stress, we considered examples where endophytes have demonstrated beneficial effects on agricultural plants, and we critically analysed potential limits of microbe-based approach. Finally, we also briefly discussed the possible future perspectives of the use of endophytes in crop production under climate change, highlighting possible limitations and improvements.

## 2 The climate change scenario and plant-microorganisms interactions

Plants, in their evolutionary path outside the oceans, have established important relationships with various microorganisms, such as bacteria, fungi, protists, and viruses (Plett and Martin, 2018). These microorganisms can live associated with different plant tissues and organs and form the plant microbiota. This can be divided in different microbial communities, based on the plant parts they colonize: the phyllosphere, which includes all the aboveground plant tissues (*i.e.*, stems and leaves), the endosphere, represented by the internal tissues, the spermosphere, *i.e.* the seed, and the rhizosphere, which comprehends the roots surface and the soil surrounding it, reached by the root exudates (Johnston-Monje and Raizada, 2011; Levy et al., 2018; Sharma et al., 2020). The different conditions of each of these habitats lead to diverse microbial communities, even within the same plant.

Through millions of years, pathogenic, competing, mutualistic, or symbiotic associations have been established between plants and microorganisms. Fossil records provide substantial evidence that over the past 450 million years, virtually all plants have formed symbiotic relationships with microbes since their first colonization of land. Various studies have documented microbial symbionts in fossilized plant specimens dating back to this era (Redecker et al., 2000; Krings et al., 2007; Bonfante and Genre, 2010; Kawaguchi and Minamisawa, 2010; Genre et al., 2020). Partida-Martínez and Heil (2011) suggested that the plant microbiota plays additional essential roles in phenotypic and epigenetic plasticity, as well as in the continuous evolution of plants.

The interactions between plants, soil, and microbes have played and continue to play a vital role by influencing various processes that contribute to plant health and productivity (Ahmed et al., 2019; Khatoon et al., 2020). Endophytes, the microorganisms of the endosphere, can provide benefits to the whole plant, either by promoting plant growth (Johnson et al., 2003; Rodriguez et al., 2008), eliciting the production of metabolites and useful chemicals such as antibiotics and agrochemicals (Kaul et al., 2012; Kusari et al., 2014; Wang et al., 2015), or helping plants to cope with stresses (Yan et al., 2019; Chen et al., 2021).

Since the first alerts of incoming human-driven climate change (Driga and Drigas, 2019), it became clear that the phenomenon would also impact the relationships between plants and microorganisms (Classen et al., 2015; Singh et al., 2019). Climate change manifests mainly as a global increase in temperature, dry periods, rapid changes of meteorological conditions (*e.g.*, flash droughts), rainfall intensity and uneven distribution (Easterling et al., 2000; Hammond et al., 2012), with differences in impact linked to various geographical regions (Surjan et al., 2016). All these deviations from a previously more stable climate, particularly the rise in temperature, are shifting plant phenology and the global distribution of plants (Sykes, 2009; Geissler et al., 2023), and significantly increase the threats to survival of natural environments (Abbass et al., 2022).

Agricultural systems are both the subject of climate change impacts, more negative than positive (Shahzad et al., 2021; Yadav et al., 2021), and contributors to it, like other human activities that require energy inputs (Hatfield et al., 2020). In fact, although on a smaller scale per unit area compared to other human activities, due to the vast extent of agricultural systems, they can contribute to GreenHouse Gases (GHGs) emissions.

Some agricultural practices, such as intense tillage, irrigation and extensive fertilizers usage, in addition to the ever-increasing use of machinery operated by fossil fuels, lead to increased emissions of GHGs in the atmosphere (Lin et al., 2011; Galic et al., 2019). As for CH_4_ and N_2_O, agricultural activities are responsible for around half and three-quarters of all anthropogenic emissions, respectively (Lynch et al., 2021). Agriculture, especially in the form of livestock production and rice cultivation, is one of the main sources of CH_4_, which has a Global Warming Potential (GWP) much stronger than CO_2_ (Saunois et al., 2020). As for N_2_O, agriculture remains the main source of this GHG, primarily through the use of nitrogen fertilizers, both synthetic and natural. The lifetime of N_2_O in the atmosphere is about 120 years, and its GWP is about 210 times higher than that of CO_2_ (Singh, 2000).

Today, converting conventional agriculture into a sustainable, yet high-yielding system, is crucial to meet both our future food needs and the integrity of the biosphere. In fact, an increasing food demand cannot be met by simply improving current agricultural practices based on fossil carbon inputs, which are detrimental to the environment (Santoyo et al., 2017; Slepetiene et al., 2020). Indeed, the continuous use of fertilizers and pesticides derived from chemical synthesis has been shown to deteriorate agroecosystems, reduce soil biodiversity, and impair natural predators of insects (McLaughlin and Mineau, 1995; Alavaisha et al., 2019).

For this reason, it was recently proposed that the deployment of plant-microbe interactions be used as one of the strategies for converting agricultural systems from the traditional mechanistic approach to the new paradigm of agroecological intensification.

## 3 The plant microbiota

The metabolism and morphology of plants and their microbiota are intrinsically connected, with a dynamic interplay between both, maintaining the function of the holobiont. The holobiont concept suggests a new perspective of organisms as meta-organisms, composed of a host organism and its associated microorganisms, co-evolved as species assemblages (Berg et al., 2016). In the plant kingdom, microbiota fulfil important functions for the holobiont, promoting its growth and increasing tolerance against biotic and abiotic stressors, as well as for the ecosystem, decomposing crop residues and contributing to nutrient cycling (Chaparro et al., 2012; Mendes and Raaijmakers, 2015; Vandenkoornhuyse et al., 2015).

The composition and functional diversity of the plant microbiota is influenced by biotic factors including age or developmental stage, species or cultivar, and plant health, as well as by abiotic factors such as soil properties, nutrient status, and climatic conditions (Berg and Smalla, 2009).

Three main classes of microorganisms are reviewed as part of the plant microbiota: endophytic fungi and bacteria, residing inside the plant tissues; rhizospheric microorganisms, residing in the soil surrounding plant roots; and mycorrhizal fungi.

### 3.1 Endophytic bacteria and fungi

An endophyte is a microorganism that lives, at least for a portion of its life cycle, inside plant tissues without producing any symptoms of disease. Among endophytic microorganism are bacteria, fungi, actinomycetes, and viruses (Bao and Roossinck, 2013; Stępniewska and Kuźniar, 2013), and they are found in almost every plant (Partida-Martínez and Heil, 2011). Endophytes can be classified as systemic (or true endophytes) or non-systemic (or transient endophytes), depending on their life cycle and the type of relationship they establish with the plants. Indeed, there is a huge variability of symbiotic lifestyles, from mutualism to parasitism, depending on genotypic and/or environmental factors. True endophytes co-evolved with their hosts, creating mutualistic relationships, and are often vertically transmitted, while transient endophytes could shift from a pathogenic to a mutualistic behaviour, depending on external conditions (Wani et al., 2015). In any case, several factors may influence the host response to endophytic interactions: mainly host genotype, nutrient availability, environment, field management practices, and microorganism strain (Hesse et al., 2003; Malinowski and Belesky, 2006; Qawasmeh et al., 2012).

Endophytes can be found in the root tissues, where they are more abundant, but also in the aerial parts of the plant (leaf, flower) and in the seeds (Hallmann, 2001).

Endophytic bacteria could be considered as a subgroup of the rhizospheric bacteria, that acquired the ability of colonizing their host plants (Reinhold-Hurek and Hurek, 1998). In fact, rhizosphere is a highly competitive environment (Raaijmakers et al., 2002), while the internal tissue of the host may represent a protected ecological niche with minor perturbations from the external conditions of the soil or, in general, of the environment (Kasmir et al., 2011), and this could have created an evolutionary drive from the first to the second. Endophytic and rhizospheric bacteria implement very similar strategies to promote plant growth, but usually endophytic microorganisms have a higher beneficial potential.

Among bacterial endophytes, *Proteobacteria* are the most widely represented, including α-, β-, and γ-*Proteobacteria*; the latter taxon being the most diverse and widespread (Miliute et al., 2015; Santoyo et al., 2016). Other classes frequently isolated from plant tissues are *Actinobacteria*, *Bacteroidetes*, and *Firmicutes* (Reinhold-Hurek and Hurek, 2011). Rarer, but still present, are *Acidobacteria*, *Planctomycetes* and *Verrucomicrobia* (Santoyo et al., 2016). The most common bacterial genera are *Bacillus* (*Firmicutes*), and *Pseudomonas* (*Proteobacteria*). *Rhizobia* spp. are also included among endophytic bacteria, as they colonize internal root tissues of *Fabaceae*, developing the typical nodules for nitrogen fixation.

Among endophytic fungi, on the other hand, we can find the families *Clavicipitaceae* (associated with grasses), *Cladosporiaceae*, *Glomerellaceae*, *Sebacinaceae*, *Pleosporaceae*, and *Hypocreaceae*, among which the most representative genus is *Trichoderma* (Hardoim et al., 2015).

Key molecular and metabolic pathways at the base of host-microbe recognition and strain selection by different plant genotypes are starting to be elucidated. It is clear how different genotypes grown in different soil/environmental combinations are enriched with different endophytic strains (Granér et al., 2003; Rashid et al., 2012; Edwards et al., 2015). The role of plant genotype on strain selection and microbial population composition has been widely studied and even if it is not the main force driving microbial diversity, it is able to modulate it (Weinert et al., 2011; Bulgarelli et al., 2015; Walters et al., 2018). Interestingly, it has been shown that plant domestication, and lately the development of high-yielding genotypes, caused a reduction in the plant capacity of associating with useful microorganism (Porter and Sachs, 2020; Valente et al., 2020), since human-centered breeding neglected the traits related to microbiota association.

Considering endopythes role in promoting plant growth, especially in nutrient-deprived conditions, and in increasing plant defence against pathogen attack, either directly or indirectly, they are now considered a tool for crop management. These could sustain agricultural practices with fewer chemical inputs. However, research is still needed to further the knowledge both on the plant-side, trying to identify the genetic factors responsible for a more efficient microbial colonization, and on the microbic-side, to isolate the most promising micro-organisms and the most effective synthetic communities. Once these aspects are clarified, it will be possible to engineer plants and microbes to make their interaction more effective. It will also be possible to explore the use of root exudates or organic compounds that might serve as pre-biotics. Finally, we should be able to overcome and the bottleneck, as of the applicability of this research to open fields conditions, that remains challenging.

Another relevant application of this category of microorganisms is phytoremediation. It has been shown that some bacterial strains are tolerant to high concentrations of heavy metals, as Cd, Cu and Zn, and other pollutants. These strains favour their accumulation into the plants, promoting their growth (Ullah et al., 2015; Ma et al., 2016), even in a stressful environment.

### 3.2 Mycorrhizal fungi

Mycorrhizae are likely to have played a crucial role in the evolution of terrestrial plants (Bonfante and Genre, 2008; Partida-Martínez and Heil, 2011). Today, mycorrhizal fungi can be divided into ectomycorrhizae, when the hyphae colonize the root intercellular spaces, and endomycorrhizae, when they penetrate inside the plant cells. Endomycorrhizae are further divided into orchid (OM), ericoid (ERM) and arbuscular mycorrhizae (AMs) (Bonfante and Genre, 2010; Chen et al., 2021).

Ectomycorrhizae are mostly associated with woody perennial trees such as *Pinaceae*, *Fagaceae*, *Dipterocarpaceae* and *Caesalpinoidaceae*, contributing to the wellness of most forest ecosystems (van der Heijden et al., 2015). EM fungi are phylogenetically diverse and belong to *Basidiomycetes*, *Ascomycetes* and *Zygomycetes*, representing the orders *Pezizales*, *Agaricales*, *Helotiales*, *Boletales*, and *Cantharellales*. EM hyphae grow partially inside the root intercellular space and partially outside, creating a mantle covering the tip of colonized lateral roots, called the Hartig net (Shi et al., 2023).

EM fungi live in symbiosis with their hosts, but are also facultative saprotrophs, decomposing complex organic matter present in the soil and making nitrogen and phosphate available for the plants (Martin and Nehls, 2009). In turn, the EM fungi receive photosynthates from the plant. Genomic data and functional studies show the presence of specialized families of phosphate, ammonium, and nitrate transporters (Jargeat et al., 2003; Casieri et al., 2013; Becquer et al., 2014; Stuart and Plett, 2019), supporting evidence of their fundamental role in different genomes of EM fungi. The entire metabolic chain transporting N/P from the soil to the plants through EM hyphae has been fully elucidated in recent years, and many advances have been made thanks to high-quality genomic sequences available (Nehls and Plassard, 2018).

Among endomycorrhizae, Arbuscular Mycorrhiza (AM) are the most represented, as these symbioses are formed by the 70-90% of terrestrial plants species, while the fungi all belong to the monophyletic *phylum Glomeromycota* (Schüβler et al., 2001). AM fungi are obligate biotrophs, considered organisms with no or rare sexual reproduction, and present aseptate hyphae developing inside the plant cells, where they form the characteristic tree-shaped hyphal structure. In AM symbiosis, the fungi support the plants by supplying mainly P-based nutrients and water, while the plant supplies the fungi with carbon nutrition (Parniske, 2008). It is estimated that almost 20% of the photosynthetic products of terrestrial plants are allocated to AM (Bago, 2000). The N contribution is less pronounced in AM compared to EM, even though some publications have shown that the portion of N transported to the plant cells from AM is not negligible and depends on soil pH, moisture, and nutrient concentration (Govindarajulu et al., 2005; Jin et al., 2005; Tanaka and Yano, 2005).

Typically, a soil ecosystem characterized by mycorrhizal symbiosis features a wide variety of plant-fungi relationships, thus offering a broad functional diversity (van der Heijden et al., 1998; Burleigh et al., 2002). This diversity arises from the presence of different plant species and fungi, each with the potential to select the most cooperative partner (Werner and Kiers, 2015). Plant roots tend to be enriched with fungal species or isolates that ensure optimal growth benefits (Bever et al., 2009; Kiers et al., 2011; Verbruggen et al., 2012), while AM fungi typically select plants that can allocate the highest amount of C nutrients (Lekberg et al., 2010; Kiers et al., 2011). However, the genetic and ecological mechanisms underlying this partner selection remain unclear, and understanding them could reveal crucial insights for future agricultural applications.

The success of the mutualistic relationship depends on many factors, such as the combination of plant-fungi genotypes, their molecular and metabolic regulation, and soil characteristics (pH, structure, moisture), nutrient availability, and colonization rate. Different plant genotypes, under controlled conditions, may respond differently to AM in terms of plant growth and yield, as shown in crops such as maize (*Zea mays* L.) (Ramírez-Flores et al., 2019) and sorghum (*Sorghum bicolor* (L.) Moench) (Watts-Williams et al., 2019), or in terms of stress resistance, as demonstrated in rice (*Oryza sativa* L.) (Chareesri et al., 2020) and bread wheat (*Triticum aestivum* L.) (Lehnert et al., 2018). The results of these studies represent milestones for future breeding programs, supporting more sustainable agriculture.

Besides AM, endomycorrhizae are also represented by ericoid (ERM) and orchid (OM) mycorrhizae. As for ERM, the fungi colonize plants of the *Ericaceae* family, such as *Calluna*, *Vaccinium* and *Erica*, typically found on nutrient-poor and acidic soils (Soudzilovskaia et al., 2019).Thus, they represent an essential way to mobilize organic material in infertile soils. In OM, the fungi belong to *Basidiomycetes*, mainly to *Rhizoctonia* species, with which most orchids are associated (Favre-Godal et al., 2020). Orchids strongly depend on the nutrients coming from the fungi, especially for the initial stages of seed germination and growth.

It should be noted that there are also plants that establish different types of mycorrhiza, either spatially, temporally, or simultaneously, within the same root system. For example, this is the case with plants from *Populus*, *Fraxinus* and *Eucalyptus* genera (Ambriz et al., 2010; Teste et al., 2020), as well as other plants known as dual-mycorrhizal plant species. It must be noted that there are also plants establishing different types of mycorrhiza, in a spatially or temporally distinguished manner, or simultaneously, within the same root system. For example, this is the case of plants from *Populus*, *Fraxinus* and *Eucalyptus* genera (Ambriz et al., 2010; Teste et al., 2020) and other plants that are called dual-mycorrhizal plant species.

It is worth mentioning that often a single fungus may connect the root systems of several plants, creating what are known as common mycorrhizal networks (Figueiredo et al., 2021). This facilitates the exchange of signalling compounds and nutrients, increases pathogen resistance, and promotes plant growth.

## 4 Plant-endophyte interactions

**Figure 1:**
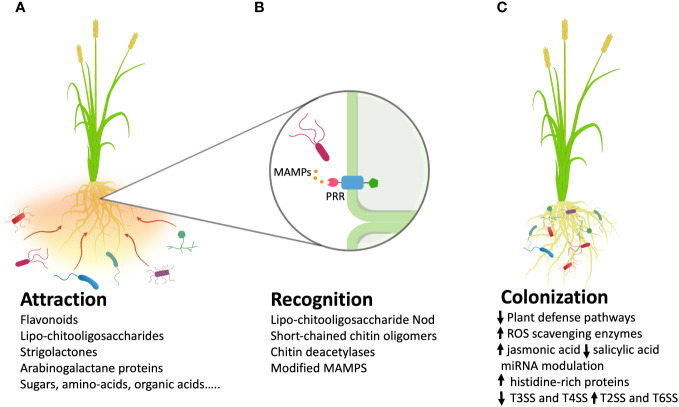
Metabolites and processes involved in three crucial steps of plant roots colonization by bacterial endophytes. **(A)** Release of molecular exudates from the roots favors the chemotactic response by the endophytes presents in the surrounding soil; **(B)** The recognition step is a complex phase in which plant receptors recognize microbial molecules that trigger molecular pathways. A typical recognition mechanism acts through MAMPs (Microbe-Associated Molecular Patterns) and plant PRRs (Pattern Recognition Receptors). **(C)** Once inside the plant, endophytes can influence many processes, for example modulating the levels of phytohormones or increasing ROS (Reactive Oxygen Species)-scavenging enzymes. (MAMPS, Microbe-Associated Molecular Patterns; ROS, Reactive Oxygen Species; T3SS, type III secretion system; T4SS, type IV secretion system; T2SS, type II secretion system; T6SS, type VI secretion system; arrow up, up regulated metabolites/processes; arrow down, down regulated metabolites/processes).

Successful endophyte colonization involves compatible plant-microbe interactions (Khare et al., 2018). Several steps can be identified to accomplish the whole process, which includes attraction, recognition, and colonization (**Figure 1**).

### 4.1 Attraction

Some endophytes are seed-borne and are present in germinated plants, thus representing a bridge across host plant generations (Coombs and Franco, 2003). Also, plants with vegetative propagation can transmit their endophytic microbiota to the next generation (Kamran et al., 2022). In others, a chemotactic response of endophytes to host plant root exudates has been observed. These exudates are rich in biomolecules (including nutrients and water), and thus attract or are recognized by friendly endophytic microbes (Compant et al., 2010; Brader et al., 2014). Flavonoids are one such group of metabolites secreted by several plants and categorized as chemo-attractants, playing an important role in endophytic interaction with the root hair. Flavonoids are used in bioformulations to affect successful infection of legume roots by rhizobia (Arora and Mishra, 2016). They are also reported to play a role with non-rhizobial endophytes, and it has been proven that in the presence of these metabolites, the colonization of roots in rice and wheat by the endophytic *Serratia* sp. EDA2 and *Azorhizobium caulinodans* ORS571 is far more effective (Webster et al., 1998; Balachandar et al., 2006). Lipo-ChitoOligosaccharides (LCO), also called Nod factors, are well-characterized signal molecules activating the Common Symbiotic Pathway (CSP) in rhizobia-legume and arbuscular mycorrhizal associations (Gough and Cullimore, 2011). Recently, StrigoLactone (SL) secreted by roots of *Arabidopsis thaliana* was found to act as a signal molecule for colonization of endophytic *Mucor* sp (Rozpądek et al., 2018). SL treatment may also activate the synthesis and release of short-chain chitin oligomers, whose perception by the plant can stimulate the symbiotic signalling pathway during early stages of host colonization (López-Ráez et al., 2017). Additionally, ArabinoGalactan Proteins (AGPs), which are highly glycosylated members of the Hydroxyproline-Rich GlycoProteins (HRGPs) superfamily of plant cell wall proteins, play a crucial role in establishing the interaction of plant with microbes (including endophyte) at several stages (Nguema-Ona et al., 2013). Several other root exudates, including sugars, amino acids, organic acids, phenolic compounds, and other secondary metabolites, are now known to be secreted by plant roots, which selectively invite the mutualistic microbes, particularly the endophytes (Chagas et al., 2017). A bacterial endophyte can also utilize the hyphae of a fungal pathogen to gain access from the soil to plant roots, thereby protecting the host from infection (Palmieri et al., 2020). showed that the endophytic rhizobacterium *Rahnella aquatilis* utilizes hyphae of the fungal pathogen *Fusarium oxysporum* to access and colonize plant roots. Metabolomic and multi-omics approaches, as those described below, would most likely increase the knowledge about metabolites released by plant seeds and roots involved in attracting favourable endophytic microorganisms. This information is necessary to address the realization of bioformulations or genetic engineering approaches to increase the production of chemo-attractants and thus the colonization of plant tissues by bacterial endophytes under normal and stressful plant growth conditions (Arif et al., 2020).

### 4.2 Recognition

The strategies that plants use to distinguish beneficial microbes, such as endophytes, from pathogens, are still a matter of research and not completely understood. Plants possess various PRRs (Pattern Recognition Receptors) that recognize M/PAMP (Microbe/Pathogen-Associated Molecular Patterns) ligands and initiate immune reactions (Hacquard et al., 2017). The most characterized MAMPs include flagellin, elongation factor Tu, peptidoglycan, lipopolysaccharides, bacterial cold shock proteins, bacterial superoxide dismutase, Beta-Glycan, β-glucans from oomycetes, and chitin (Newman et al., 2013). These MAMPs are recognized on the surface of plant cells by PRRs, which include receptor-like kinases and receptor-like proteins (Tang et al., 2017). Both pathogens and symbionts can be recognized by PRRs, because the M/PAMPs are not specific to pathogens. To avoid recognition by the host plant and the subsequent immune response, pathogens and symbionts have evolved complex extracellular invasion strategies. Due to the similarity of pathogen and symbiont genomes (Reinhardt et al., 2021), common extracellular strategies exist between them. They can be divided into three categories: avoiding accumulation of MAMP precursors, reducing hydrolytic MAMP release, and preventing MAMP perception (Buscaill and van der Hoorn, 2021). These strategies can involve different microbial effectors. Symbionts have developed various strategies to allow their potential hosts to better distinguish them from pathogens during the recognition phase. For example, LCO Nod Factors are perceived by legumes, activating the symbiotic pathway (Radutoiu et al., 2003; Bozsoki et al., 2020). In rice, short-length chitooligosaccharide (CO4) triggers symbiotic signal transduction with the symbiotic complex receptor MYR1–CERK1.This suppresses the formation of the CEBiP-CERK1 heteromer that would mount the immune response, while long-chain chitooligosaccharide (CO8) induces immune signalling through CEBiP-CERK1 (Chiu and Paszkowski, 2021; Zhang et al., 2021). It has also been observed that fungal endophytes produce chitin deacetylases, which deacetylate chitosan oligomers that are thus not perceived by plant receptors (Cord-Landwehr et al., 2016). There is also evidence where endophytic bacteria are known to produce their own MAMPs, which are either not recognized by PRRs of plants or trigger in plants a comparatively weak and transient defence reaction compared to pathogenic interactions (Vandenkoornhuyse et al., 2015). Along this line, it was shown that in grapevine (*Vitis vinifera* L.), thanks to an alteration in sequence, the perception of flagellin from an endophytic *Burkholderia phytofirmans* by LRR-RLK (Leucine-Rich Repeat-Receptor-Like Kinase) FLAGELLIN-SENSITIVE 2 (FLS 2) PRR was different from the perception of those of bacterial pathogens, such as *Pseudomonas aeruginosa* or *Xanthomonas campestris* (Trdá et al., 2015). Still, there are knowledge gaps about the genetic mechanisms that differentiate recognition strategies deployed by beneficial with respect to pathogenic microbes, that need to be filled through comparative genomic studies between the two microbial categories, complemented with functional analyses. Availability of complete information on gene functions involved in the endophytic recognition would allow targeted modifications of favourable strains through gene editing and/or over-expressing approaches that would improve the plants capability in recognizing microbial symbionts and protecting them from the immune response.

### 4.3 Colonization

Potential entry points for endophytes are cracks formed at the emergence of lateral roots, zones of root elongation, root hair cells, and wounds. Other sources include stomata, particularly of young stems and leaves, lenticels, and germinating radicles. For successful colonization, some bacteria must find their way to these apertures. *Klebsiella pneumoniae* 342 (Kp342) can colonize the lateral root junctions in wheat and alfalfa (*Medicago sativa* L.) (Dong et al., 2003). Similarly, *Herbaspirillum seropedicae* and *Gluconacetobacter diazotrophicus* dominate colonization at lateral root junctions (James et al., 1997; Luna et al., 2012). Some endophytes enter through infection colonization, where cellulolytic and pectinolytic enzymes produced by endophytes come into play (Miliute et al., 2015), such as pectate lyase, which has been implicated in the colonization of *Klebsiella* strains (Kovtunovych et al., 1999). Symbionts can colonize hosts while overcoming the response to Damage-Associated Molecular Patterns (DAMPs) and MAMPs, while a response against pathogens is still possible in the presence of non-pathogenic microbes (Zhou et al., 2020). Several studies have proven that there is a downregulation of plant defence pathways during the colonization of plants by mutualistic partners, such as rhizobia or AMF (Fouad et al., 2014; Benhiba et al., 2015). In the case of an oxidative burst or generation of Reactive Oxygen Species (ROS) as plant defence system, endophytes protect themselves by producing enzymes such as superoxide dismutases, catalases, peroxidases, alkyl hydroperoxide reductases, and glutathione-S-transferases (Zeidler et al., 2004). The root endophytic fungus *Serendipita indica* secretes a histidine-rich protein to improve its access to micronutrients and to influence oxidative stress and reactive oxygen homeostasis to facilitate the colonization of the host plant (Nostadt et al., 2020). Also, symbionts could induce Jasmonic Acid (JA) and suppress Salicylic Acid (SA) formation to Induced Systemic Resistance (ISR), whereas pathogens typically enhance the SA biosynthesis to mediate Systemic Acquired Resistance (SAR) in plants (Martínez-Medina et al., 2017). Moreover, during mutualistic interactions, late induction of SA/JA/ET signalling pathways prevents the microbe from ‘overstepping’ and ‘overpowering’ the plant (Plett and Martin, 2018). It is reported that most miRNAs induced in the host during the establishment of endophytes also target hormone-response pathways (Formey et al., 2014). During AMF infection, the miRNA E4D3Z3Y01BW0TQ is upregulated and disrupts Gibberellic Acid (GA) signalling pathway, known for repressive action against mutualistic associations (Formey et al., 2014; Martín-Rodríguez et al., 2015; Wu et al., 2016). The plant may also induce the expression of different groups of genes during colonization by diverse sets of microbes. For example, during the establishment of symbiosis, the majority of pathways targeted by miRNAs for plant defence system are turned off, thus preventing the obstacle to the proliferation of endophytes (Plett and Martin, 2018). For AMF, two receptor-like kinases called Arbuscular Receptor-like Kinase 1 (ARK1) and ARK2 are required for the sustenance of the symbiotic interaction in several plant species (Montero et al., 2021). Moreover, AMF are separated from the plant cytoplasm by a specialized host-derived membrane, which represents the main interface facilitating the bidirectional exchange of nutrients and information and protects the microbial symbionts from the immune response (Huang et al., 2021). The biosynthesis of this peri-arbuscular membrane is controlled by a gene called *GLUCOSAMINE INOSITOL PHOSPHORYLCERAMIDE TRANSFERASE1 (GINT1)* (Moore et al., 2021). Protein Secretion Systems (SSs) in bacteria also modulate the plant immune system. Among all known SSs, Type III Secretion System (T3SS) and Type IV Secretion System (T4SS) are essential for delivering Effector Proteins (EFs) by the pathogenic bacteria into the plant, but these are either absent or present in low abundance in mutualistic endophytic bacteria (Green and Mecsas, 2016; Liu et al., 2017). Notable exceptions can be seen in some rhizobial strains where T3SS is important for nodulation of some legumes (Ausmees et al., 2004; Okazaki et al., 2016, 2013). The T3SS is also a determinant for rice endophyte colonization by non-photosynthetic *Bradyrhizobium* spp (Piromyou et al., 2015). Furthermore, the Type 2 Secretion System (T2SS) was demonstrated to be required for suppressing MAMP-triggered immunity in efficient root colonizer bacteria and, notably, enhanced the colonization capacity of other tested commensal bacteria in *Arabidopsis* (Teixeira et al., 2021). On the other hand, in mutualistic proteobacterial endophytes, Type VI Secretion Systems (T6SSs) are present, and are also commonly found in commensal and pathogenic plant-associated bacteria. However, they are associated with important functions, which are apart from virulence, usually such as competition against other bacteria (Reinhold-Hurek and Hurek, 2011; Bernal et al., 2018). From this picture, it emerges that colonization involves a plethora of traits from both, plants and microorganisms and available data most likely shed light only on a small fraction of the involved processes. Considering the plant side, in addition to the information provided above, recent investigations highlighted that plant genes can shape the microbiota composition (Zhang et al., 2019; Deng et al., 2021; Escudero-Martinez et al., 2022; Oyserman et al., 2022; Escudero-Martinez and Bulgarelli, 2023) and that wild germplasm is supposed to support higher microbiome diversity than domesticated counterparts (e.g (Pérez-Jaramillo et al., 2018; Nerva et al., 2022). Taken together, these results indicate that there is room for genetics interventions addressed to increase both plant and beneficial microbial aptitude in establishing favourable interactions and that further functional and multi-omics investigations can increase the available targets for improving endophytic colonization by plant growth promoting microorganisms. Once plants and microbial effective targets are identified, these could be modified/introgressed/engineered into their respective genomes (Arif et al., 2020; Nerva et al., 2022; Escudero-Martinez and Bulgarelli, 2023).

## 5 Omics for the study of plant-endophyte interactions

The intricate network of interactions among the various actors of the microbiota requires the use of advanced techniques with higher likelihood of obtaining global information from the organisms. This is to decipher a complex system and attempt to clarify the role of each organism at the genetic, transcriptional, metabolic, and physiological/phenotypic level.

The microbiota consists of several microorganisms inhabiting soil layers and distinct plant tissues (Bulgarelli et al., 2013; Compant et al., 2019), among which different relationships can be established, depending on environmental factors. A multi-layer communication web organizes the connections among the microorganisms, between the different plants growing in the same soil, and between plants and microorganisms (Hassani et al., 2018; Xiong et al., 2021). Much has been learned about these mechanisms in recent years, thanks to the advent of Next Generation Sequencing (NGS) and, more broadly speaking, to the “omic” technologies, *i.e.* genomics, transcriptomics, proteomics, and metabolomics. The plant-microbe scientific community has greatly benefited from them.

### 5.1 Genomics and metagenomics

The development of NGS technologies has allowed to perform whole genome sequences of numerous fungi and bacteria. Overcoming the limit of traditional culture-dependent identification approaches, it has enabled the identification of as much microbial diversity as possible. Meta-genomic approaches nowadays almost routinely make use of DNA extraction from the whole soil/tissue microbial population, allowing the analysis of its gene/taxa content using next generation sequencing (Allan, 2014). The sequencing can involve the whole genome, which is then tentatively assembled and annotated, or only the 16S rRNAs. These data can be used to study the microbial diversity and to evaluate the absolute abundance of different bacterial strains, taking into account the different copy number of 16S rRNA genes in distinct bacterial genomes (Case et al., 2007; Wang et al., 2020).

It is worth mentioning that the availability of several AM fungi genomes has allowed for the study of the evolution of these organisms, which are considered as living fossils and ancient asexuals (Parniske, 2008). Their genome size is highly variable, from the 39.6 Mb of *Paraglomus occultum* (Malar C et al., 2022) to 784 Mb of *Gigaspora margarita* (Venice et al., 2020), with large genomes hosting a higher number of genes and a high proportion of transposable and active elements (Venice et al., 2020; Dallaire et al., 2021); differences that could explain their intra-specific variability.

In parallel, the study of the epigenome variability is emerging as a tool to understand a hidden layer of variability (Chaturvedi et al., 2021). Another interesting example of recent scientific advances given by the most recent sequencing technologies concerns the use of long-read sequencing and chromatin conformation capture techniques that made it possible to understand the genomic organization of multi-nucleate coenocytic hyphae of AM, demonstrating their heterokaryotic nature and supporting rare sexual reproduction events (Yildirir et al., 2022; Sperschneider et al., 2023).

### 5.2 Transcriptomics

This approach, coupled to advanced bioinformatic pipelines, for example using algorithms of artificial intelligence, could be considered as the most useful omic science for understanding the network of interactions. It has largely benefited from NGS technologies, whose recent advances have significantly increased the sensitivity to catch the rarest transcripts. Moreover, long-read sequencing technologies in the Iso-Seq approach, among others, allow to cover the entire transcript length thereby distinguishing rare isoform resulting from alternative splicing events (Li et al., 2017; Zhang et al., 2019).

Transcriptomics has been successfully applied to uncover the plant molecular strategies used to recruit the most favourable microbial organisms in response to diverse abiotic and biotic stresses, and to understand the microbial molecular networks used to successfully establish the symbiotic relationships (Sheibani-Tezerji et al., 2015). Furthermore, transcriptomic studies applied to bacterial cells may help decipher which strains and cells, among the endophytic or rhizospheric population, are transcriptionally active (Sharma et al., 2004; Knauth et al., 2005; Sheibani-Tezerji et al., 2015), surmounting DNA-based technologies that cannot distinguish non-viable cells. The completion of whole genome sequencing of new microbial species and strains will be crucial allowing the identification of the microbial response to different soil characteristics and plant genotypes.

RNA-seq has also been applied to the population of small RNAs, to identify and characterize plant miRNAs involved in host-microbiota communications. These small non-coding RNAs are important key regulators of different plant biological pathways, from organ development to stress response. It has been shown that microorganisms might stimulate the expression of plant miRNAs, modulating drought tolerance response, nutrient uptake, or facilitating symbiosis establishment (Mohsenifard et al., 2017; Pentimone et al., 2018; Kord et al., 2019; Tao et al., 2023). Besides plant miRNAs, other small RNA-like molecules are coded by fungi and bacteria, that could be involved in an intriguing system of cross-kingdom RNAi-mediated regulation, for example during mycorrhizal colonization (Silvestri et al., 2019), or nodule formation (Ren et al., 2019). The intriguing hypothesis of small RNAs as mobile cell-to-cell signalling molecules (Huang et al., 2019) has been explored in detail thanks to the possibility to purify Extracellular Vesicles (EV). In fact, EVs have been shown to transport small RNAs between plant host and microorganisms in both pathogenic and mutualistic interactions (Cai et al., 2019), thus their further analysis will deepen our understanding of below-ground inter- and intra-kingdom communications. Single cell transcriptomics, coupled with enhanced microscopy techniques will greatly improve our understanding of endophyte bacteria and AM fungi lifestyle inside the plants (Yin et al., 2023) and of root cells regulatory network.

### 5.3 Proteomics and metabolomics

In parallel with next-generation and third-generation (or single molecule) (Schadt et al., 2010) sequencing technologies, which have been successfully applied to the study of all the DNA/RNA populations present in a tissue, the analyses aimed at characterizing the entire set of proteins, with their post-translational modifications, and metabolites have evolved. This evolution is to comprehensively study all the molecules in a microbe/plant biological system, thereby increasing their sensitivity and throughput. Proteomics has been applied to plant tissues to understand how the presence of an endophyte, for example, may modulate the synthesis of different plant proteins (Lery et al., 2011), revealing their role in cellular recognition. The analysis of the protein-protein interactions, also called an interactome, is essential to unveil molecular mechanisms at the base of symbiotic relationships.

Metabolomics and proteomics have been used to analyse root exudates, containing both primary and secondary metabolites, to understand how biotic and abiotic factors might modulate their composition, and as a result, attract and associate with different microorganisms. However, analysing either the metabolites or the proteins present in a colonized plant tissue, or both, is challenging. This is because it is difficult to distinguish between molecules produced by either the plants or the fungi/bacteria. Recently, to resolve this issue, several techniques have been developed to narrow the analyses to the single-cell level, such as Mass Spectrometry Imaging (Boughton and Thinagaran, 2018), Laser ablation electrospray ionization (Bhattacharjee et al., 2020), live single-cell mass spectrometry (Masuda et al., 2018), and the spatial metabolomics pipeline (Geier et al., 2020).

In addition to soluble metabolites, plants can diffuse Volatile Organic Compounds (VOCs).Metabolomics is essential to uncover the role of these signalling molecules and their modulation in response to environmental stimuli and genotype interactions. However, their role in soil matrices could be less abundant and relevant than in aerial open-air environments.

Metabolomic analyses have shown that plants can influence their microbiota by secreting various metabolites. In turn, the microbiome can influence the metabolome of the host plant (Haichar et al., 2008).

It is now clear that to acquire global information on the interconnections existing among plants and the microbiota, single omics technologies should be integrated into a multi-omics approach (Chen et al., 2021). To this end, the development of bioinformatic tools and networking models that can integrate and visualize information is essential. This will provide a comprehensive view of the regulatory network connecting all the molecular levels from the genome to the metabolic pathways. By employing a multi-omics approach, it will be possible to deepen our knowledge on the complex interactions between plants and their growth-promoting microbial counterparts. This will be fundamental to understand how to engineer microbial communities and plants for a more sustainable agriculture. In this scenario it is fundamental to develop high-throughput phenotyping platforms, to measure and analyze qualitative and quantitative traits on a large scale, developing suitable phenomics approaches, that could be non -invasive and able to work in the field as well, in order to fill the gap with other omics techniques (Großkinsky et al., 2015).

## 6 The role of endophytes in protection from abiotic stresses

Plants, as sessile organisms, face continuous exposure to environmental stresses. These include both biotic factors, such as pathogens, pests and herbivores, and abiotic factors, such as heat, cold, drought, salinity, waterlogging, heavy metal toxicity, nutrient deficiency, and oxidative stresses (Cramer et al., 2011). Climate change has been increasing the negative effects of these abiotic stresses, leading to both faster events of severe stresses (e.g., flash droughts (Pendergrass et al., 2020)), and to more prolonged periods of stress, with several negative impacts on plant growth and productivity, up to more than -50% (Mahajan and Tuteja, 2005; Lohani et al., 2020; Sandrini et al., 2022).

Abiotic stress like heat or cold extremes can cause changes in membrane fluidity and protein structure, while the presence of salt or heavy metals in the soil can alter the physiological processes of enzymes and molecular interactions (Zhang et al., 2022). Salinity can also negatively impact the photosynthetic components, reducing the assimilation of CO_2_ and the absorption of light. This, in turn, can lead to an increase of ROS and oxidative stress (Ma et al., 2020). It is important to consider that heat, drought, and salt stress are commonly present together, exacerbating the detrimental effects on plants. To sense and respond to abiotic stresses, plants have evolved multiple complex mechanisms, which have been extensively reviewed in the last decades (Zhang et al., 2022).

**Figure 2:**
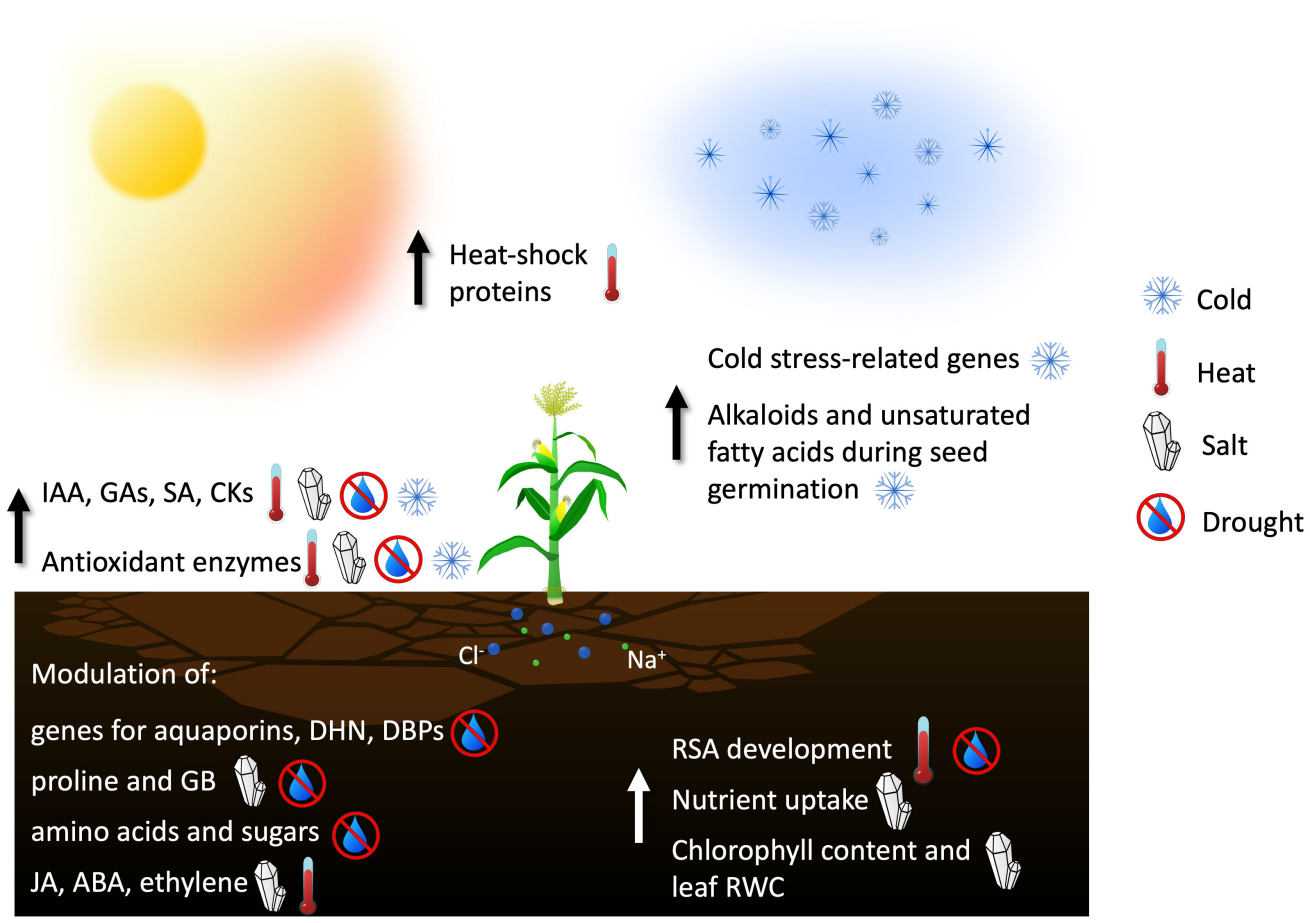
Main contributions of endophyte microorganisms in enhancing plant tolerance to abiotic stresses through increasing the synthesis of antioxidant molecules and heat-shock proteins, modulating the amount of phytohormones, or stimulating the development of the root structure (IAA, Indole-3-Acetic Acid, indicating auxins in general; GAs, Gibberellins; SA, Salicilic Acid; CKs, Citokinins; DHN, Dehydrin family of proteins; DBPs, Dehydration responsive element Binding Proteins; GB, Glycine Betaine; JA, Jasmonic Acid; ABA, Abscissic Acid; RSA, Root Structure Architecture; RWC, Relative Water Content).

Considering this negative scenario, plant-associated microorganisms appear to be promising allies for modern agriculture to face climate change (**Figure 2**; **Table 1**). For example, many rhizobacteria produce osmoprotectants in the presence of stress conditions, while other bacteria, like *Pseudomonas* spp., produce ExoPolySaccharides (EPS) to increase water retention in case of drought stress (Rathinasabapathi, 2000; Grover et al., 2011). The mechanisms of both interactions and potential advantages exploitable in agriculture are reviewed for each stress type.

**Table 1:** Overview of mechanisms of protection of plants from abiotic stresses by beneficial endophytes.

A)				

Endophyte Species	Host plant	Increased Tolerance to Stress	Mechanisms	References
		Single Stress		
*Epichloe gansuensis*	Drunken horsegrass (*Achnatherum inebrians*)	Cold	Increase alkaloids biosynthesis and unsaturated fatty acids during seed germination	Chen et al., 2016
*Piriformospora indica*	Maize (*Zea mays* L.)	Drought	enhance the expression of genes involved in the drought stress response of maize hosts by increasing auxin, ABA, SA, and cytokinin levels	Zhang et al., 2018
*Trichoderma harzianum*	Rice (*Oryza sativa*)	Drought	Modulate activity of genes for aquaporin and dehydrin, dehydration responsive element binding protein, and SuperOxide Dismutase	Pandey et al., 2016
*Piriformospora indica*	Barley (*Hordeum vulgare*)	Drought	Increase the production of high temperature stress-responsive proteins	Ghaffari et al., 2019
AMF culture (mainly *Rhizophagus intraradices*; *Funneliformis mosseae*; *F. geosporum*)	Wheat (*Triticum aestivum*)	Drought	Increase of reachable soil water thanks to hyphae	Mathur et al., 2019
*Porostereum spadiceum* AGH786	Soybean (*Glycine max*)	Salt	Decrease of JA and ABA, increase of GA3	Hamayun et al., 2017
*Yarrowia lipolytica* FH1	Maize (*Zea mays* L.)	Salt	Secretion of exogenous IAA and regulation of endogenous IAA and ABA. Effects on production of peroxidase, catalase and proline	Gul Jan et al., 2019

B)				

Species	Host plant	Increased Tolerance to Stress	Mechanisms	References
		Single Stress		
*Burkholderia phytofirmans* PsJN (PGPR)	Grapevine (*Vitis vinifera* L.)	Cold	Induction the up-regulation of cold stress-related genes	Theocharis et al., 2012
*Brevibacterium linens* RS16	Rice (*Oryza sativa* L.)	Heat	Reduction of ethylene levels, enhancement in GST expression, increased levels of small HSPs	Choi et al., 2022
*Bacillus aryabhattai* SRB02	Soybean (*Glycine max*)	Heat	Increase in phytohormones production, modification of stomata behaviour and root structure	Park et al., 2017
*Streptomyces* sp. PGPA39	Tomato (*Solanum lycopersicum*)	Salt	Decrease of ethylene through ACC deaminase production	Palaniyandi et al., 2014
		Multiple Stresses		
*Paraburkholderia phytofirmans* PsJN (PGPR)	Thale cress (*Arabidopsis thaliana*)	Heat and Drought	Enhancement in the development of RSA	Macabuhay et al., 2022
*Bacillus* spp.	Fennel (*Foeniculum vulgare* Mill.)	Drought and Salt	Increase of availability of P	Mishra et al., 2016

A): Fungi; B): Bacteria.

B).

### 6.1 Temperature

Temperature changes are not necessarily harmful to plants; they can play a crucial physiological role in regulating internal clocks and controlling processes like the opening and closing of flower corollas. Some species require exposure to low temperatures to initiate important developmental processes, such as vernalization for flowering or germination (Ruelland and Zachowski, 2010). However, temperatures that are too low can induce a range of physiological responses that can be detrimental to their survival. Chilling, intended as a few degrees above 0°C air temperature, can cause reductions in enzymatic activity, rigidification of membranes, destabilization of protein complexes, and stabilization of RNA secondary structure, while also promoting the accumulation of ROS. Additionally, chilling can impair photosynthesis and increase membrane permeability, resulting in the leakage of cellular contents. Freezing (below 0°C) stress, on the other hand, can cause more severe damage, as ice formation within cells leads to mechanical disruption and cell/tissue/plant death (Ruelland and Zachowski, 2010).

There are not many reports available in literature about the protective effects of endophytes against the low temperature stresses, perhaps because such conditions also limit the growth and multiplication of microorganisms. As an example of protective effects towards cold stress, the endophytic fungus *Epichloe gansuensis* increases the biosynthesis of alkaloids and unsaturated fatty acids during the seed germination of Drunken horsegrass (*Achnatherum inebrians*), thereby increasing tolerance to cold stress (Chen et al., 2016). It was also reported that the endophytic rhizobacterium *Parabulkholderia. phytofirmans* PsJN induced the upregulation of some cold stress-related genes in grapevine (Theocharis et al., 2012; Chen et al., 2021).

Heat stress is defined as a temperature rise of 10-15°C above ambient, with the optimal range for plant growth about 15-24°C. Among all the different abiotic stress factors, it has the most detrimental effects on plants, reducing flower fertility and modifying crop growth with a detrimental final effect on yield (Shaffique et al., 2022). Its effects include an increase in membrane fluidity, the formation of ROS, and alterations to photosynthesis and respiration processes (Banerjee and Roychoudhury, 2018; Shaffique et al., 2022). Heat stress triggers a cascade of physiological responses that result in the release of Heat Shock Proteins (HSPs), a class of molecular chaperones that facilitate protein folding and prevent aggregation under conditions of cellular stress. These proteins can assist unfolded proteins in refolding into their proper conformation or direct them towards degradation through ubiquitination processes (Ruelland and Zachowski, 2010; Banerjee and Roychoudhury, 2018). The often simultaneous presence of heat and drought stress exhibits holistic features, as the combined effects are greater than those caused by each stress individually (Lipiec et al., 2013).

The inoculation of the endophytic bacterium *Brevibacterium linens* RS16 in rice plants reduced the emission of the plant stress hormone ethylene due to its 1-aminocyclopropane-1-carboxylate (ACC) deaminase activity. Plants inoculated with *B. Linens* RS16 also showed increased levels of small HSPs (Choi et al., 2022).

A recent report demonstrates that the application of the plant growth-promoting root endophyte *Paraburkholderia phytofirmans* PsJN enhances the development of the Root System Architecture (RSA) in *Arabidopsis thaliana*, under both normal and high-temperature conditions. This allows the plant to access a larger soil area, thereby better managing abiotic stresses such as heat and drought (Macabuhay et al., 2022).

The simultaneous presence of heat and salinity stress can greatly impact crops. The inoculation of the endophytic fungus *Trichoderma virens* SB10, along with Glycine Betaine (GB) treatments, conferred significant tolerance in soybean (*Glycine max* L.) plants against these two stresses. In presence of GB, *T. virens* SB10 enhanced the production of hormones like gibberellins, IAA, and SA. The co-treatment with the fungus and GB also led to a reduction in proline accumulation and Na^+^ uptake and an increase in macronutrient (N, Ca, K) uptake. Effects on *GmHKT1* and *GmSOS1* gene expression, two major genes involved in salt tolerance (Singh and Roychoudhury, 2021), were also recorded, leading to the maintenance of a high K^+^/Na^+^ ratio. Treated plants exhibited higher growth rates and an increase in antioxidant activities due to the upregulation of Ascorbate PeroXidases (APX), SuperOxide Dismutases (SOD), PerOXidases (POD) and reduced Glutathione (GSH) enzymes (Bilal et al., 2023).

### 6.2 Drought and salinity

These two stresses represent the main abiotic stress factors that limit crop production globally (Trenberth et al., 2014; Rodriguez and Durán, 2020).

Drought is defined as a period when the available water is insufficient for an organism or environment to function at its best (Kamran et al., 2022). Drought stress represents one of the most critical threats to plant productivity, and thus to global food production, affecting all the stages of plant growth. It leads to a reduction in turgor pressure, affecting cell division, enlargement, and differentiation (Farooq et al., 2009, 2009).

Drought thus affects plants in many ways: typical symptoms include stunted plants, scorching, rolling, and yellowing of leaves, and permanent wilting (Seleiman et al., 2021). Moderate drought stress can induce modifications in the RSA and in the allocation of resources by the plant. In the case of severe drought stress, the roots shrink, and alterations occur at PhotoSystem II (PS II) (Ma et al., 2020).

Soil salinity is defined as the increased amount of sodium (Na^+^) and especially chloride (Cl^-^) ions in soils, resulting primarily from natural events such as weathering of parent rocks, seawater, or atmospheric deposition. Other than that, anthropogenic processes, such as poor drainage facilities, irrigation with brackish groundwater, unsuitable water management, and ‘cultural’ errors in irrigated agriculture, can increase soil salinity (Evelin et al., 2019). Salinity causes ionic imbalance and alters metabolic pathways in plant cells, like the synthesis of proteins and the function of some enzymes and ribosomes. Besides, Na^+^ competes with other essential nutrients like phosphate, nitrate and potassium (Angon et al., 2022).

From several reports, it became evident that the plant microbiome can play a role in protecting against high salinity and drought (Yang et al., 2009; Rolli et al., 2015; Berg et al., 2016).

The endophytic fungus *Piriformospora indica* enhances the expression of genes involved in the drought stress response of maize hosts by increasing auxin, ABA (ABscissic Acid), SA, and cytokinin levels (Zhang et al., 2018). Also, *Trichoderma harzianum* was shown to improve drought tolerance in rice, by modulating the activity of genes for aquaporin and dehydrin, Dehydration responsive element-Binding Protein (DBP), and SOD (Pandey et al., 2016).

Symbiotic relationships between plants and endophytic fungi such as *Piriformospora indica* can enhance the adaptation of plants to drought stress by regulating amino acid and soluble sugar metabolism. For instance, *P. indica* was found to improve the adaptation of barley (*Hordeum vulgare* L.) to drought stress (Ghaffari et al., 2019). Soybean inoculated with the endophytic fungus *Porostereum spadiceum* AGH786 under saline conditions showed reduced effects of salinity. The endophyte caused decreasing levels of JA and ABA and increasing levels of GA3, leading to improved plant growth (Hamayun et al., 2017). Similarly, researchers observed a positive effect of the interaction between the endophytic fungus strain *Yarrowia lipolytica* and maize under saline conditions, which improved plant growth attributes such as leaf relative water content, levels of oxidative enzymes and chlorophyll content through the enhancement of metabolism and hormones (ABA and IAA) secretions (Gul Jan et al., 2019).

Endophytic microbes can alleviate the salt-generated oxidative stress in plants by activating genes for ion transporters, ROS scavenging, and by activating the production and signalling of phytohormones such as auxin, JA, and Ethylene (ET) (Brotman et al., 2013; Ghaffari et al., 2016; Qin et al., 2018; Eida et al., 2019).

Finally, seed bio-priming is a novel beneficial technique aiming to employ bio-stimulating agents like growth-promoting microorganisms to improve the physiological functioning of seeds and their stress resilience (Chakraborti et al., 2022). Two salt-tolerant endophytic fungi, *Paecilomyces lilacinus* KUCC-244 and *Trichoderma hamatum* Th-16 were used for bio-priming wheat and mung bean (*Vigna radiata* L.) seeds. Results showed that both endophytes, in particular *T. hamatum* Th-16, increased the growth and chlorophyll content of wheat and mung bean plants under extreme salinity conditions. The primed plants also exhibited increased activities of antioxidant enzymes and enhanced photosynthetic attributes (Irshad et al., 2023).

## 7 The role of endophytes in biotic stresses tolerance

Biotic stress occurs when the plant is damaged by phytopathogens, such as bacteria, fungi, viruses, insects, or herbivores that feed and thrive at the plant’s expense. It is the primary cause of harvest losses, especially those caused by bacterial and fungal phytopathogens (Chaudhary et al., 2022). It is estimated that biotic stresses cause approximately 17-30% of global crop production loss (Muthu Narayanan et al., 2022).

Until now, the standard procedure to combat plant pathogens infection has been the use of chemicals. This mechanistic approach often does not consider any ecosystemic interaction. However, pesticides can be hazardous, and the occurrence of pesticide resistance is another significant factor to consider (Hawkins et al., 2019).

Against these types of stresses, a great deal of research activity and farm-scale applications of beneficial organisms are reported, much more than against other crop limitations, such as abiotic stresses. Biological control represents a promising strategy to manage plant pathogens sustainably. The first uses of insect parasites date back to the end of the 19^th^ century (Hajek et al., 2007). Biological control involves applying either beneficial organisms, or substances produced by them, such as enzymes, phytohormones, and secondary metabolites, to alleviate the negative effects caused by pathogens and stimulate favourable reactions in the plant (Muthu Narayanan et al., 2022).

**Figure 3:**
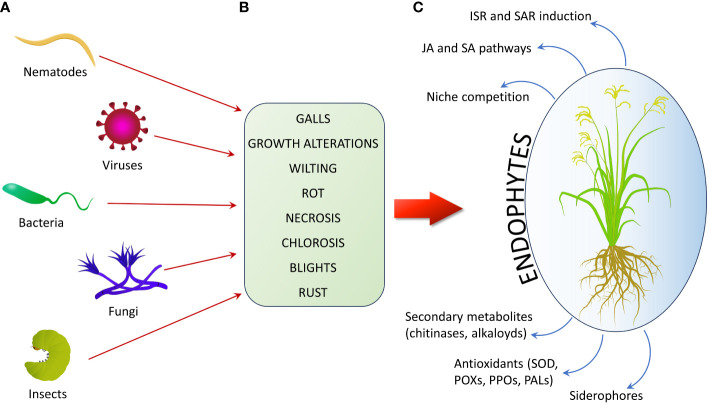
Schematic example of plant damages caused by phytopathogens and relative mechanisms by which endophytes can help crops contrasting pests. **(A)** Main groups of plant pathogens. **(B)** Principal types of damages caused by phytopathogens. **(C)** Main plant defense responses enhanced by endophytes to contrast plant parasites (ISR, Induced Systemic Resistance; SAR, Systemic Acquired Resistance; JA, Jasmonic Acid; SA, Salicylic Acid; SOD, Super-Oxide Dismutase; POX, PerOXidase; PPO, PolyPhenol Oxidase; PAL, Phenylalanine Ammonia Lyase).

Endophytes can use direct mechanisms to exert their biocontrol effects against phytopathogens, including the production of siderophores, to limit the availability of metal ions to pathogens, or the synthesis of antifungal compounds, or competition for a biological niche. They can also counteract pathogens through indirect mechanisms, by inducing SAR and ISR in the host plant (Pandey et al., 2019; Kamle et al., 2020; **Figure 3**; **Table 2**).

**Table 2:** Overview of mechanisms of protection of plants from biotic stresses (plant pathogens, pests, and parasites) by beneficial endophytes.

A)				

Pathogen/Pest	Endophyte species	Host Plant	Effects	Mechanisms	References
Bacteria					
*Ralstonia solanacearum*	*Trichoderma harzianum*	Tomato (*S. lycopersicum* L.)	Destruction of bacterial cells	Release of fungal metabolites	Yan and Khan, 2021
Viruses					
Tobacco Mosaic Virus (TMV)	*Aspergillus versicolor*	Tobacco (*Nicotiana glutinosa* L.)	Moderation of anti-TMV activity	Synthesis of aspernolides C and D butyrolactones	Zhou et al., 2015
Maize Chlorotic Mottle Virus (MCMV) and SugarCane Mosaic Virus (SCMV)	*Trichoderma harzianum; Metarhizium anisopliae*	Maize (*Zea mays* L.)	Reduction of the pathogenic effects of SCMV (*T. harzianum*); Decrease in titer of SCMV (*M. anisopliae*); No evident effects on MCMV	Not yet defined, but possibly due to activation of defence-related genes and the interference exerted by endophytes on virus movement	Kiarie et al., 2020
Pests/Parasites					
*Meloidogyne javanica* (Nematode)	*Trichoderma harzianum* BI	Tomato (*S. lycopersicum* L. var. Roma VF)	Reduction of nematode eggs hatching, increase of antioxidant enzymes	Penetration inside the nematode egg mass matrix	Sahebani and Hadavi, 2008
*Meloidogyne incognita* (Nematode)	*Trichoderma harzianum* T-78	Tomato (*S. lycopersicum* L.)	Reduction of nematode eggs clusters, delay in the development of eggs	Enhancement of JA- and SA-regulated defences	Martínez-Medina et al., 2017
Insects and herbivores	*Clavicipitaceae*	(The paper does not focus on a specific species of plant)	Feeding deterrence, delayed development, increased mortality	Production of bioactive alkaloids like ergot, indole-diterpenes, lolines, peramine	Panaccione et al., 2014

B)				

Pathogen	Endophyte species	Host Plant	Effects	Mechanisms	References
Fungi					
Botritys cinerea	*Micromonospora* spp.	Tomato (*S. lycopersicum* L.)	Reduction in the infection rates	Increased induction of JA-related pathways	Martínez-Hidalgo et al., 2015
Pyricularia oryzae	*Bacillus* spp.	Rice (*Oryza sativa* L.)	Increase of antioxidant response, reduction of blast disease symptoms	Increased synthesis of antioxidant enzymes, secretion of proteases, glucanases, siderophores	Rais et al., 2017
Fusarium graminearum	*Streptomyces* strain DEF09	Spring wheat (*Triticum aestivum* L.)	Inhibition of fungal spreading from the infection site	Chitin degradation, synthesis of antifungal molecules	Colombo et al., 2019
Rhizoctonia solani	*Ochrobactrum ciceri* SR_1_EB_1;_ *Achromobacter spanius* SR_1_EB_11;_ *Bacillus licheniformis* SR_2_EB_5_	Stevia (*Stevia rebaudiana* Bertoni)	Growth inhibition of hyphae	Stripping of fungal hyphae and accumulation of debris	Vyas and Singh, 2023
Bacteria					
*Pectobacterium* sp.	*Streptomyces* sp. TP199	Potato (*S. tuberosum* subsp. *Tuberosum* L.*)*	Reduction of tubers tissue maceration	Synthesis of antimicrobial compounds, interference on communication signals	Padilla-Gálvez et al., 2021
Pests/Parasites					
*Meloidogyne javanica* (Nematode)	*Streptomyces* strain SA	Banana (*Musa acuminata* AAA Cavendish)	Inhibition of nematodes	Synthesis of antibiotics effective against nematodes (avermectin, nanchangmycin, milbemycin), niche competition	Su et al., 2017

A): Fungi; B): Bacteria.

### 7.1 Bacterial pathogens

Phytopathogenic bacteria are predominantly represented by the genera *Agrobacterium*, *Bacillus*, *Burkholderia*, *Clavibacter*, *Erwinia*, *Pantoea*, *Pseudomonas*, *Ralstonia*, *Streptomyces*, *Xanthomonas*, and *Xylella* (Muthu Narayanan et al., 2022). Bacterial diseases can be systemic or localized, with the most common symptoms in plants being galls and overgrowth, wilting, rot, scabs, necrosis, chlorosis, and blights (Nazarov et al., 2020).

Fungal metabolites produced by *Trichoderma harzianum* have shown strong antibacterial activity against *Ralstonia solanacearum*, a phytopathogenic bacterium that causes bacterial wilt disease in tomato (*Solanum lycopersicum* L.) plants (Yan and Khan, 2021).

Endophytic actinobacteria were isolated from Chilean native potatoes (*Solanum tuberosum* subsp. *tuberosum* L.) and they were demonstrated to act against *Pectobacterium carotovorum* subsp*. Carotovorum* and *P. atrosepticum*, bacterial pathogens that cause tissue maceration symptoms in potato tubers. One of the isolates, *Streptomyces* sp. TP199, was found to inhibit the growth of *Pectobacterium* sp., reducing tissue maceration symptoms. Moreover, strain TP199 showed metal-dependent Acyl Homoserine Lactones (AHL) quorum quenching activity, which can inhibit communication between bacterial cells (Padilla-Gálvez et al., 2021).

### 7.2 Fungal pathogens

The most common plant pathogenic fungi are *Alternaria* spp., *Aspergillus* spp., *Colletotrichum* spp., *Fusarium* spp., *Phytophthora* spp., *Pythium*, and *Pyricularia* spp., while anthracnose, dieback, gall, powdery mildew, blight, rust, rot, wilt, and smut are examples of diseases caused by these fungal phytopathogens (Muthu Narayanan et al., 2022).

Some *Bacillus* strains significantly increased antioxidant enzymes like SOD, PerOXidase, PolyPhenol Oxidase and Phenylalanine Ammonia Lyase in leaves and roots of the rice plant, contrasting the fungus *Pyricularia oryzae*. They also secreted different biocontrol molecules such as proteases, glucanases, and siderophores in the rice rhizosphere (Rais et al., 2017). In wheat, *Fusarium graminearum* is the cause of Fusarium head blight as well as Fusarium foot and root rot. Colombo and colleagues (Colombo et al., 2019) studied the biocontrol activity of *Streptomyces* spp. on *F. graminearum* in spring wheat and one strain, DEF09, effectively inhibited FHB under controlled and field conditions by blocking the spread of the pathogen at the infection site.

Endophytes with biocontrol potential against *Rhizoctonia solani*, a fungal pathogen causing sheath blight disease in maize, were isolated from *Stevia rebaudiana* plants. Three bacterial isolates, identified as *Ochrobactrum ciceri* SR1EB1, *Achromobacter spanius* SR1EB11 and *Bacillus licheniformis* SR2EB5, showed growth inhibition effects against *R. solani* (Vyas and Singh, 2023).

Furthermore, the gram-positive bacterium *Micromonospora*, isolated from nitrogen-fixing nodules of leguminous plants, showed biocontrol effects on fungal pathogens and stimulation of plant immunity in tomato. Root inoculation with *Micromonospora* strains showed reduced infection from the fungal pathogen *Botrytis cinerea*, and investigations on defence mechanisms highlighted an increased induction in JA-related defence pathways (Martínez-Hidalgo et al., 2015).

### 7.3 Viral pathogens

Plant viruses are globally diffused plant pathogens, obligatory parasites due to their need for replication within plant cells. Plant viruses are primarily RNA viruses, and the ones considered most important are Tobacco Mosaic Virus (TMV), belonging to the family *Virgaviridae*, Tomato Spotted Wilt Virus (TSWV) (*Tospoviridae*), Tomato Yellow Leaf Curl Virus (TYLCV) (*Geminiviridae*), Cucumber Mosaic Virus (CMV) (*Bromoviridae*), Potato Virus Y (PVY) (*Potyviridae*), Cauliflower Mosaic Virus (CaMV) (*Caulimoviridae*), African Cassava Mosaic Virus (ACMV) (*Geminiviridae*), Plum Pox Virus (PPV) (*Potyviridae*), Brome Mosaic Virus (BMV) (*Bromoviridae*), Potato Virus X (PVX) (*Alphaflexiviridae*) (Scholthof et al., 2011). Symptoms of viral diseases in plants include growth suppression, deformation, discoloration, necrosis, and impaired reproduction (Nazarov et al., 2020). Investigating the contribution of microbiota towards viral infection (Zhou et al., 2015), identified two new butyrolactones (aspernolides C and D) along with two previously known butyrolactones (aspernolides A and B) from a culture of the endophytic fungus *Aspergillus versicolor*. When tested against viruses, both aspernolides C and D exhibited moderate anti-TMV activity. Similarly, Kiarie et al. (2020) tested the ability of fungal endophytes to contrast Maize Lethal Necrosis (MLN), a serious disease affecting maize crops in eastern Africa. This disease is caused by the co-infection of maize plants with Maize Chlorotic Mottle Virus (MCMV) (*Tombusviridae*) and Sugarcane Mosaic Virus (SCMV) (*Potyviridae*). Maize plants inoculated with *T. harzianum* and *Metarhizium anisopliae* showed reduced severity and titer of SCMV, respectively, indicating their potential to induce resistance against SCMV. However, no significant effect was observed on the MCMV.

### 7.4 Insects and herbivores

Invasive insects cause at least $70 billion in crop losses every year (Bradshaw et al., 2016). Panaccione et al. (2014) summarized a series of studies on the role of bioactive alkaloids produced by endophytic fungi in protecting plants against herbivores. Four major types of bioactive alkaloids (ergot alkaloids, indole-diterpenes, lolines and peramine) are produced by fungi from the *Clavicipitaceae* family. Symbioses between plants and these endophytes have significant effects on both insects and mammalian herbivores, largely due to the production of these bioactive alkaloids. Ergot alkaloids contribute to herbivore resistance, also affecting nematodes. They also act through feeding deterrence, delayed development, and increased mortality of insects.

Loline alkaloids exhibit insecticidal and feeding-deterrent activity. Lolines are often present in grasses with fungal endophytes of the genera *Epichloë* and *Neotyphodium* (Wilkinson et al., 2000). Genetic analyses to determine whether the production of lolines in plants is active against aphids highlighted that the endophyte *Epichloë festucae* showed heritable variation in the expression of loline alkaloids. Analyses on Lol+ (alkaloid expression) and Lol- (no expression) linked alkaloid expression to activity against aphids, and the levels of alkaloids in the plants were correlated with the level of anti-aphid activity (Wilkinson et al., 2000).

Peramine is the most widely distributed of the four classes of *epichloae*-derived secondary metabolites. It is another alkaloid that acts as a strong feeding deterrent for different insects. Peramine is water-soluble and is dispersed throughout the plant (Rowan, 1993; Panaccione et al., 2014).

Additional information about the role of Endophytic EntomoPathogenic Fungi (EEPFs) was recently made available by (Samal et al., 2023).

On the other hand, different endophytes could be used not only to prevent herbivore damage in plants but, in some cases, to favour this phenomenon for domestic herbivores, reducing undesirable molecules present in plants for livestock nutrition (Bluett et al., 2005a, 2005b).

### 7.5 Nematodes

Nematodes are small, non-segmented invertebrates. They are the most abundant animals on Earth and are fundamental to the soil-food web (Gamalero and Glick, 2020). The phylum *Nematoda* comprises more than 30,000 species (Bernard et al., 2017), classified into five groups: bacterivores, fungivores, herbivores, omnivores, and predators (van den Hoogen et al., 2020).

Nematodes include the so-called Plant-Parasitic Nematodes (PPN), among which some of the most important are root-knot nematodes (*Meloidogyne* spp.), cyst nematodes (*Heterodera* spp. and *Globodera* spp.), and root-lesion nematodes (*Pratylenchus* spp.) (Kumar and Dara, 2021). Over 4,100 species of PPNs have been identified, causing an estimated $80–$118 billion dollars per year of damage to crops (Bernard et al., 2017). PPNs can damage the host plant through a needle-like oral structure called stylet, used to release specific enzymes inside plant tissues (Pulavarty et al., 2021, p. 202), and the group of root-knot nematodes develop root knots by forming a complex of multinucleate hypertrophied giant cells, which cause visible knots or galls at the root level (Martínez-Medina et al., 2017). More detailed information about nematodes, their characteristics, and modes of action can be found in Jones et al. (2011).

The main way to fight PPNs is to use chemical nematocides, but these are expensive and harmful to the environment, and EU regulations are constantly reducing the nematocides available for agriculture (Poveda et al., 2020). Therefore, the biocontrol of nematode infection is becoming a promising possibility.

Endophytes isolated from banana (*Musa acuminata* AAA Cavendish) plant roots infected with *Meloidogyne* spp. were tested against *Meloidogyne javanica*, and one strain, named SA and identified as *Streptomyces* spp., showed an inhibition rate of more than 50% *in vitro* and a biocontrol efficiency of more than 70% in sterile soil against the nematode (Su et al., 2017).

Fungi could also represent a valuable source of biocontrol agents against plant-infecting nematodes. The fungus *Trichoderma harzianum* strain BI was used against *M. javanica* (Sahebani and Hadavi, 2008), reducing the infection rates of the nematode through penetration inside the nematode egg mass matrix, leading to reduced hatch levels. *T. harzianum* BI also increased the activity of resistance-related enzymes in plants, such as POX, PPO, and PAL. Further investigation showed that chitinase activity in *T. harzianum* BI culture filtrates increased in the presence of colloidal chitin and nematode eggs, implying its potential for degradation of chitin present in nematode eggs.

The root endophyte strain *T. harzianum* T-78 was used to study the protective effects on tomato plants against the root-knot species *M. incognita* (Martínez-Medina et al., 2017) using a split-root system, in which the two halves of the plant roots were allowed to grow in two different pots, one for the treatment and the second as a control. T-78 inoculation decreased the amount of root galls, and a significant reduction in the number of nematode egg clusters was observed in systemic tests. Moreover, the expression profile of the SA-responsive marker genes Pathogenesis-Related protein 1a (PR1a) and Pathogenesis-Related protein P6 (PR-P6) was higher in T-78-pretreated plants infected with the nematode and SA concentrations were higher compared with the non-pretreated ones. Finally, the expression analysis of the JA-responsive genes Proteinase Inhibitors II (PI II) and MultiCystatin (MC) after *M. incognita* infection showed that JA signalling was down-regulated in plants not inoculated with T-78, while in tomato plants pre-inoculated with T-78, the inhibition in genes PI II and MC showed a significant reduction.

More specific reviews are available to deepen the knowledge about the use of endophytes as biocontrol agents against nematodes (e.g., Gamalero and Glick, 2020; Kumar and Dara, 2021).

## 8 Role of endophytes in nutrition and quality of the final products

Plant–microbe interactions play a crucial role in improving soil nutrition and enriching micronutrients through metal solubilization, mobilization, and translocation to different parts of the plant. Micronutrient deficiency, also known as “hidden hunger”, threatens the health of billions of people worldwide, particularly in developing countries. Additionally, the intensification of crop production is causing a gradual depletion of micronutrients in agricultural soils, compromising the nutritional value of food. Iron and zinc deficiencies are widespread in the human diet, leading to several malnutrition symptoms. To overcome these deficiencies, biofortification, the process of increasing the bioavailable concentrations of essential elements in the edible portion of crop plants, is commonly achieved through plant breeding and agronomic practices. Microbial communities can be exploited as a valid alternative due to their ability to increase metal solubilization in the soil and enhance their mobilization to the plant parts. This is achieved through the production of siderophores or other chelating factors, upregulation of Zn and Fe transporters, acidification of the rhizosphere through organic acid secretion and proton extrusion, reduction of anti-nutritional factors (e.g. phytic acids), and secretion of phenolics or phytohormones like signalling molecules (Singh and Prasanna, 2020). Both bacteria and fungi have demonstrated a positive effect in improving mineral contents in the edible parts of plants, although a major representation of mycorrhizal fungi underlines the importance of this category of endophytes for supporting plant nutrition.

Bacterial endophytes have proven to be effective in the biofortification of wheat grains with Zn (Ramesh et al., 2014; Abaid Ullah et al., 2015; Singh et al., 2017). Wang et al. (2014) found a positive influence of rice inoculation with an endophyte recovered from Zn hyper-accumulator *Sedum alfredii* on the bioavailability of Zn in the soil and its accumulation in rice grains. Vaid et al. (2014) tested the effect of zinc-solubilizing bacteria on growth promotion and zinc nutrition of rice: bacterial inoculations significantly enhanced the total Zn uptake as well as grain methionine concentration, besides increasing the mean of many agronomic traits, like dry matter yield, productive tillers/plant, number of panicles/plants, number of grains/panicle, grain and straw yield. The screening of 129 strains of endophytic bacteria from maize stem and leaves showed that 24.5% of these isolates were siderophore producers, 14% could solubilize insoluble Zn compounds and 33% of them had phytase activity (Verma et al., 2022). Rana et al. (2012) reported a significant increase in Fe, Mn, and Cu content in wheat grains upon inoculation with the bacterial strain *Providencia* spp. isolated from the wheat rhizosphere. The screening of a set of 213 endophytes from several wheat genotypes allowed Singh et al. (2018) to identify promising endophytes for Zn and Fe accumulation in wheat grains. At the same time, in grains after endophyte inoculations, phytic acid, an anti-nutritional factor, was significantly decreased.

Subramanian et al. (2013) reported that the inoculation of maize plants with AM fungi improved the availability of micronutrients in soils, particularly Zn, as a consequence of rhizospheric acidification and siderophore production, and produced grains with 10-15% higher Fe and Zn contents, while the anti-nutritional factor phytic acid decreased. In wheat, the application of a consortium of AMF resulted in an increase of micronutrients (Cu, Fe, Zn, Mn) and macronutrients (N, P, K) content in the grain (Mäder et al., 2011). AMF were also able to improve selenium (Se) level in the grain, alone or in association with selenobacteria (Duran et al., 2013). Tang et al. (2022) focused on the effects of endophytic fungus *Phomopsis liquidambaris* on the absorption and distribution of 14 essential mineral elements in the vegetative organs and in grains of rice: the results indicated that *P. liquidambaris* significantly increased the accumulation of N, P, Fe, Mn, Zn, Mo, and Se in rice grains, accompanied by a significant increase in yield and protein content. AM fungal inoculation was also effective in improving the nutritional value of chickpea (*Cicer arietinum* L.) grain by protein, Fe and Zn grain biofortification (Pellegrino and Bedini, 2014).

The interactions of endophytic fungi with plant tissues can also boost secondary metabolite production, resulting in the development of several bioactive compounds. In lettuce (*Lactuca sativa* L.), AM fungi, in addition to increasing fresh weight, improved the ascorbate level (Baslam et al., 2011). In spinach (*Spinacia oleracea* L.), they augmented the concentration of total phenolic compounds, flavonoids and phenolic acids (Khalid et al., 2017). In tomato, AMF inoculation, in addition to increasing fruit N, P, and Cu concentration, allowed for higher antioxidant concentration and carotenoid contents (Hart et al., 2015), while in strawberry (*Fragaria x ananassa* var. Selva) increased concentrations of anthocyanins (Lingua et al., 2013) and of sucrose, glucose, and two vitamins, ascorbic and folic acid (Bona et al., 2015).

Heavy metals contamination of agricultural soils is an important issue all around the world, posing serious risks to food safety. Indeed, although they are not essential elements for a plant’s life, crops uptake heavy metals in soils by root systems, they transport them to aerial parts through the xylem and the phloem, and accumulate them in edible parts, threatening the food chain, and ultimately human health. Under natural conditions, heavy metals in soils originate geologically; however, their amounts are continually increased in soils by anthropogenic sources, i.e., atmospheric deposition of Particulate Matter (PM) from industrial activity and transportation, agricultural activity, such as wastewater irrigation, the application of pesticides and fertilizers (Shi et al., 2018). Among heavy metals, cadmium (Cd) and arsenic (As) are the major contaminant in agricultural soils. Cadmium, with a biological half-life of 10-30 years, has been classified as a potent human carcinogen. Endophytes and AM fungi are involved in alleviating metal toxicity to the host plant. Bacteria evolved various mechanisms to avoid heavy metal stress including: (a) transport of metals across the cytoplasmic membrane; (b) biosorption and bioaccumulation to the cell walls; (c) metal entrapment in the extracellular capsules; (d) heavy metals precipitation; and (e) metal detoxification via oxidation–reduction (Zubair et al., 2016). Several mechanisms have been hypothesized for AMF-mediated detoxification of heavy metals, including (i) bound to cell wall and deposit in the vacuoles of AMF, (ii) sequestration by the help of siderophores in the soil or into root apoplasm, (iii) bound to metallothioneins or phytochelatins inside the fungal or plant cells, and (iv) transporters at the tonoplast of both plants and fungi catalyse the transport of metals from the cytoplasm and allow their compartmentalization into vacuoles (Jan and Parray, 2016).

Among cereals, rice plants tend to accumulate more Cd than others, and this is of particular concern in the larger rice-growing areas, where populations are relying on rice for most of their caloric intake (Hu et al., 2016). Zhou et al. (2021) identified an endophytic bacterium from Cd‐contaminated soil capable of promoting rice growth and reducing Cd concentration in rice grain under Cd‐contaminated conditions.

## 9 Conclusions and future perspectives

With a focus on endophytes, we have only scratched the surface of the enormous amount of data and information that has been produced by the scientific community worldwide, regarding the complex interactions between plants and microorganisms. Despite significant progress, many challenges remain, both from the technical and the legislative sides.

From a technical point of view, a deep understanding of the biology, way of action, and main interactions of the endophytes in the complex system of microorganism-plant-environment is necessary to optimize their usage in a one-health vision. For example, it is necessary to overcome the technical limits due to the very low fraction of culturable microorganisms inhabiting the soil, the difficulty in maintaining a stable inoculated microbial symbiont in the soil, and in increasing the plant’s aptitude to associate with beneficial microorganisms. Related to this last point, it is relevant to deepen the knowledge related to processes involved in the establishment of successful associations, namely in the attraction, recognition and colonization steps, in order to allow knowledge-driven interventions. Recent investigations uncovered, from the plant side, sorghum, tomato and barley loci affecting microbiome composition (Deng et al., 2021; Escudero-Martinez et al., 2022; Oyserman et al., 2022), thus opening the way for exploiting host-genetics to manipulate and select the crop microbiota (Escudero-Martinez and Bulgarelli, 2023), while from the microbial side new approaches of microbiome engineering that boost the positive impact of the associations are emerging (Arif et al., 2020; Nerva et al., 2022). Increasing the available targets for improving endophytic colonization by plant growth promoting microorganisms rise the possibility that in the near future it will be possible to improve plant association with beneficial microbiota through plant breeding and microbiome engineering approaches. Along these concepts is the realization of knowledge-based synthetic microbial communities (SynComs) or Artificial Microbial Consortia (AMC), that generate a defined microbial system with known taxonomic and functional profiles, thus containing multiple functions for plant growth promotion (Arif et al., 2020; Nerva et al., 2022). This can potentially solve some of the drawbacks of traditional microbial biofertilizers, such a host incompatibility, ineffective competitiveness with indigenous microbes, and inadaptability to the local environment.

Field studies provide the most natural conditions for exploring the roles of endophytes. However, the strong impact of environmental factors makes them highly variable, and often take to unpredictable results. Moreover, agricultural systems and systems intensively used by humans are often characterized by a shift (often a reduction) in microbial diversity; and this may also be extended to plants raised in pot experiments, where we expect a reduced diversity or altered structure of the microbiota (Berg et al., 2016), that pose other limits to their extension to agricultural field systems.

When transitioning from controlled experiments to field applications of potential microbial formulations, the selected strain would have to interact with naturally occurring soil microbes and endophytes. The administered endophytes should be able to colonize a broad spectrum of crops and they should acquire a niche in the plant habitat, avoiding at the same time possible negative alterations of the ecosystem. Additionally, it is also crucial to consider the proper formulation to maximize the beneficial impact on crops, while reducing costs and number of applications (Verma et al., 2021).

Another element of concern is the ability of some endophyte to become a pathogen or produce toxins. For example, fungal endophytes from genera *Fusarium*, *Alternaria*, or *Aspergillus*, possess qualities as PGPM, but are also mycotoxins producers (Stranska et al., 2022).

From a legislative point of view, in the translation from research to application, in each area of the world, the categorizations and rules imposed by current and future legislation on the microbial compounds must be taken into great account for their deployment in the real world. For example, in Europe beneficial microorganisms are divided in two main categories, owing to the target. The microbes that act against biotic targets (e.g. pathogens, pests) are defined as Biological Control Agents (BCA), are enclosed in the plant protection products, and are ruled by the EC Regulation no. 1107/2009.The microorganisms whose target is the mitigation of an abiotic stress (e.g. freezing temperatures, salinity) are defined as Microbial Plant Biostimulants (MPB), and are ruled by EC Regulation no. 1009/2009, within the category PFC 6(A).

The rapidly changing climate presents a complex and daunting challenge that requires urgent solutions, with anthropogenic causes of pollution and environmental degradation continuing to worsen rapidly despite warnings from experts. Even though association of plant with beneficial microorganisms is demonstrated to protect plants from a changing environment, it should also be considered that diverse environmental conditions, including climate changes, can produce currently unpredictable outcomes on the interactions between host plant and endophytic microbiota.

However, there is hope for a better future. As is normal in research, big changes start from small discoveries. The approaches we have explored in this review offer potential solutions to counteract the negative consequences of environmental stressors. Fortunately, ever innovating omic techniques and the ever-expanding set of genomic technologies offer powerful tools to help researchers gain deeper insights into the complex relationships between plants and their microbial partners. By using these tools, together with beneficial endophytes, to develop more eco-friendly and efficient agronomic practices, we can work towards a more sustainable future for our planet.

## Author contributions

LS: Conceptualization, Investigation, Visualization, Writing – original draft, Writing – review & editing. EM: Writing – original draft, Writing – review & editing. GV: Conceptualization, Supervision, Visualization, Writing – original draft, Writing – review & editing. PV: Writing – original draft, Writing – review & editing. NP: Conceptualization, Supervision, Writing – original draft, Writing – review & editing.
